# Beyond cell cycle control: *CDKN2A* loss is associated with altered NAD^+^ metabolic states and increased sensitivity to NAMPT inhibition in glioblastoma

**DOI:** 10.1093/noajnl/vdag088

**Published:** 2026-04-13

**Authors:** Swati Dubey, Guanqiao Yu, Ryana Aboul-Hosn, Christopher Tse, David A Nathanson, Albert Lai, Keith Vossel, Fausto J Rodriguez

**Affiliations:** Pathology and Laboratory Medicine, David Geffen School of Medicine, University of California Los Angeles, Los Angeles, California, USA; Pathology and Laboratory Medicine, David Geffen School of Medicine, University of California Los Angeles, Los Angeles, California, USA; Pathology and Laboratory Medicine, David Geffen School of Medicine, University of California Los Angeles, Los Angeles, California, USA; Department of Molecular and Medical Pharmacology, David Geffen School of Medicine, University of California Los Angeles, Los Angeles, California, USA; Department of Molecular and Medical Pharmacology, David Geffen School of Medicine, University of California Los Angeles, Los Angeles, California, USA; Department of Neurology, David Geffen School of Medicine, University of California Los Angeles, Los Angeles, California, USA; Department of Neurology, David Geffen School of Medicine, University of California Los Angeles, Los Angeles, California, USA; Pathology and Laboratory Medicine, David Geffen School of Medicine, University of California Los Angeles, Los Angeles, California, USA

**Keywords:** CDKN2A, cell cycle, Glioblastoma, Glioma, NAD^+^

## Abstract

While *CDKN2A* loss is classically associated with cell cycle deregulation through the p16-Cdk4-Rb axis, our findings suggest an additional layer of metabolic vulnerability arising from altered NAD^+^ homeostasis in *CDKN2A*-deleted glioblastoma, revealing a metabolic-genetic interface for rationally revisiting NAD^+^ targeting strategies, moving beyond the broad inhibition approaches.

Despite extensive therapeutic advances, glioblastoma (GBM) remains one of the deadliest human malignancies, with median survival rarely exceeding 18 months. One of the defining hallmarks of GBM is its extraordinary metabolic plasticity, enabling adaptation to diverse nutrient and redox environments. Distinct molecular subtypes of GBM display divergent metabolic preferences,^1^ however, the molecular circuits enabling these metabolic adaptations remain poorly defined. Among metabolic pathways supporting these adaptive programs lies nicotinamide adenine dinucleotide (NAD^+^), which fuels multiple metabolic and non-metabolic pathways, including glycolysis, oxidative phosphorylation, fatty acid oxidation, and the activity of PARPs and sirtuins.^2^^,^^3^ Early enthusiasm for targeting NAD^+^ metabolism in cancer centered on inhibition of the rate-limiting NAD^+^ salvage enzyme nicotinamide phosphoribosyltransferase (NAMPT), which is also overexpressed in GBM and correlates with an adverse clinical outcomes.^2^ Selective NAMPT inhibitors such as FK866 and KPT-9274 effectively depleted intracellular NAD^+^ and induced apoptosis across multiple tumor types, including GBM.^3^ However, dose-limiting toxicity in clinical trials dampened momentum and led to the assumption that therapeutically targeting NAD^+^ may not be feasible. Recent advances have revitalized this area. It is now clear that NAD^+^ metabolism is compartmentalized and context-dependent, with distinct regulatory mechanisms in the cytosol, nucleus, and mitochondria, and that cancer cells may rely on specific, rather than global, NAD^+^ dependencies. Recent reports, including the mitochondrial arm of NAD^+^ metabolism with the discovery of SLC25A51 as the principal mitochondrial NAD^+^ transporter, have begun to uncover new layers of complexity in NAD^+^ homeostasis and metabolic plasticity.^4^^,^^5^ In addition, a very recent study identifying gliocidine as a modulator of NAD^+^-dependent metabolism in GBM underscores a growing recognition that the NAD^+^ network is far more dynamic and context-dependent than previously appreciated.^6^ This resurgence underscores that instead of abandoning the NAD^+^ targeting strategies due to NAMPT inhibitor toxicity, selective and context-specific vulnerabilities rather than pan-cancer NAD^+^ depletion may hold the key to successful translation of NAD^+^-based therapies. Here, we revisited NAD metabolism as a therapeutic frontier in GBM, integrating transcriptomic analyses (TCGA-GBM dataset) with NAD^+^ perturbation studies using NAMPT inhibitors in GBM cell lines, and identified a link between NAD^+^ metabolic dependency and *CDKN2A* deletion, one of the most frequent genomic aberrations in GBM.

We evaluated the sensitivity of a panel of GBM cell lines to 2 distinct NAMPT inhibitors, FK866 and GNE-617. Both compounds significantly reduced cell viability (measured as resazurin-based metabolic activity) in a dose-dependent manner across GBM models (Figure 1a). In contrast, normal human astrocytes exhibited preserved viability and metabolic activity under similar conditions, indicating tumor-selective vulnerability. To provide redox-independent validation of cell viability, apoptosis analysis following 24-h FK866 treatment in U251 cells demonstrated a concentration-dependent increase in apoptotic cells, supporting reduced cell survival upon NAMPT inhibition (Figure S1). Notably, most GBM cells harboring *CDKN2A* deletion showed heightened sensitivity to NAMPT inhibition compared to *CDKN2A*-intact counterparts (Figure 1a, Tables S1-S3), which prompted us to investigate the link between *CDKN2A* status and NAD^+^ metabolism. To this, we analyzed TCGA-GBM transcriptomic datasets stratified by *CDKN2A* status. Tumors with *CDKN2A*-deep deletion showed significant enrichment of oxidative phosphorylation (OXPHOS) (Hallmark OXPHOS, NES = 2.57, *P*adj < .01) (Figure 1b), consistent with reports that RB pathway disruption drives oxidative metabolism and increases NAD^+^ demand.^7^ Then, we looked into the NAD^+^ salvage pathway. Although the overall NAD^+^ salvage pathway was not significantly enriched (REACTOME_NICOTINAMIDE_SALVAGE, NES = 1.7, *P*adj = .1), individual genes showed distinct regulation: *NAMPT* was significantly upregulated (*P* < .05), whereas *NMNAT2*, which converts NMN to NAD^+^, was downregulated (*P* < .001) in *CDKN2A*-deleted tumors (Figure 1c and f). Given the elevated OXPHOS signature, we anticipated increased mitochondrial NAD^+^ import in *CDKN2A*-deleted tumors. Surprisingly, this oxidative phenotype coincided with significant downregulation of *SLC25A51* (*P* < .001), the principal mitochondrial NAD^+^ transporter (Figure 1c). This downregulation of *SLC25A51* presents a paradoxical scenario and made us question of how *CDKN2A*-null tumors sustain high oxidative metabolism under constrained NAD^+^ import capacity. While transcript levels do not directly reflect mitochondrial NAD^+^ import capacity or compartmental NAD^+^ pools, this differential expression indicates altered regulation of NAD^+^ transport components in the *CDKN2A*-null context. One possibility is that the NAD^+^ and redox network in *CDKN2A*-deleted tumors might have rewired to sustain high OXPHOS and redox homeostasis. Interestingly, we found that this downregulation of *SLC25A51* was accompanied by upregulation of other mitochondrial transporters that play essential roles in shuttling metabolites critical for redox balance and TCA cycle activity, including (1) *SLC25A45*, associated with NMN (NAD^+^ precursor) transport and (2) *SLC25A11*, the oxoglutarate/malate carrier critical for TCA cycle flux and redox shuttling that transfers reducing equivalents between cytosol and mitochondria (Figure 1c and f).^8^^,^^9^ To determine whether the concurrent upregulation of *SLC25A45* and *SLC25A11* reflects a compensatory response to *SLC25A51* suppression independent of *CDKN2A* status, we stratified both *CDKN2A*-deleted and *CDKN2A*-intact cases by *SLC25A51* expression (top and bottom 25%) and performed correlation analyses. Interestingly, in the *CDKN2A*-deleted cohort, *SLC25A51* expression showed a modest negative correlation with both *SLC25A11* and *SLC25A45* (r ≈ −0.3), whereas no significant correlation was observed in *CDKN2A*-intact tumors (Figure 1d and e), suggesting that this compensatory rewiring is genotype-specific, rather than a general mitochondrial stress response. Since these correlations are modest, they can only be interpreted as suggestive of coordinated transcriptional association rather than evidence of functional compensation. Furthermore, *SLC25A20*, the carnitine-acylcarnitine translocase essential for fatty acid β-oxidation-driven OXPHOS, was also upregulated in *CDKN2A*-deleted tumors (Figure 1c).^10^ Unlike published findings in *SLC25A51*-null cells, we did not observe differential expression of the mitochondrial folate/FAD carrier *SLC25A32* (Figure 1c).^5^ Together, these data propose a model in which *CDKN2A*-deleted GBM cells sustain high OXPHOS despite reduced cytosolic NAD^+^ synthesis (lower *NMNAT2* expression) and constrained mitochondrial NAD^+^ import (lower *SLC25A51* expression) by compensating through alternative shuttling via *SLC25A11* and *SLC25A45* upregulation (Figure 1f). Consequently, they become acutely dependent on cytosolic NMN/NAD^+^ regeneration via NAMPT, explaining their heightened sensitivity to NAMPT inhibition. We also mapped the rate-limiting enzymes of the 2 other NAD^+^ biosynthetic pathways, including de novo/kynurenine and Preiss-Handler, within TCGA-GBM transcriptomic datasets stratified by *CDKN2A* status and found that differential expression was predominantly confined to salvage pathway genes (*NAMPT*, *NT5E*, *NNMT*), whereas de novo and Preiss-Handler pathway genes (*QPRT*, *NAPRT*) were not significantly altered (Figure S1), supporting a preferential association between *CDKN2A* loss and NAD^+^ salvage pathway dependency. Next, to functionally test this causality, we performed an isogenic *CDKN2A* knockout in GS116 cells; however, NAMPT inhibitor sensitivity was not significantly altered compared to CRISPR control (data not shown). These results indicate that while *CDKN2A* status correlates with NAMPT inhibitor sensitivity across GBM models, loss of CDKN2A alone may not be sufficient to drive this phenotype; these changes may be cell line dependent, and other genetic/epigenetic factors may contribute to the metabolic changes and pharmacologic susceptibilities observed.

**Figure 1.**
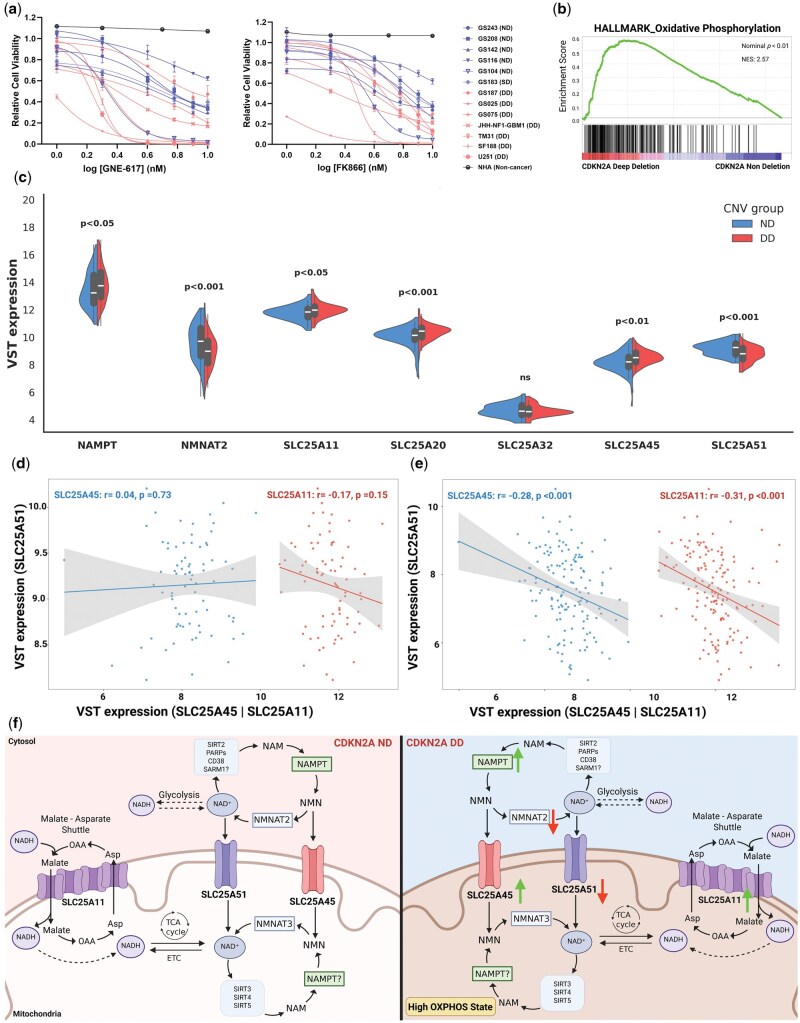
(a) Dose-response curves of GBM cell lines to NAMPT inhibitors GNE-617 and FK866. *CDKN2A*-deleted lines show heightened sensitivity than intact counterparts (*P*adj= .007-.0003 at multiple dosage levels; significance was calculated using mixed-effects models and Benjamini–Hochberg correction for pairwise comparisons; see Tables S2 and S3 for details); NHA cells (non-cancer control) remain unaffected up to 100 nM (data not shown beyond 10 nM). Data are mean ± SD (*n* = 4, ≥3 independent experiments, ND: non-deletion, SD: shallow-deletion, DD: deep deletion of *CDKN2A*); (b) GSEA of TCGA GBM data showing significant enrichment of mitochondrial oxidative phosphorylation signatures in *CDKN2A*-deleted tumors; (c) variance stabilizing transformation (VST) expression of genes by *CDKN2A* status in TCGA-GBM: non-deletion (ND, blue; copy number ≥2, *n* = 78) vs deep-deletion (DD, red; copy number = 0, homozygous deletion, *n* = 147). Gene-level comparisons were adjusted for multiple testing using FDR correction; (d and e) gene-gene correlation of *SLC25A51* expression with *SLC25A45* and *SLC25A11* by *CDKN2A* status. A modest negative correlation was observed in *CDKN2A*-deleted tumors (r ≈ −0.3) (e); while there was no significant correlation in *CDKN2A*-intact tumors (d); (f) proposed a model for a summary schematic of pathway and gene expression differences in *CDKN2A* DD and ND tumors (created in BioRender. https://BioRender.com/bf73dzj).

Overall, while our analyses provide robust correlative insights, our findings are derived from pharmacologic sensitivity assays and transcriptomic-based analyses, which support functional linkage but do not establish mechanistic rewiring of NAD^+^ pools and compartmental redox balance. However, these preliminary findings do support the emerging concept that considering both metabolic state and genetic context can improve therapeutic targeting in GBM. Fully achieving this potential will require embracing and continuing to explore the complexities of synthesis, transport, and catabolism of the NAD^+^ metabolome.

## Supplementary Material

vdag088_Supplementary_Data

## Data Availability

Data generated as part of this project will be made available upon request.

## Supplementary Material

Methods, codes and additional supporting results, are provided as supplementary information. Supplementary material is available online at *Neuro-Oncology Advances* (https://academic.oup.com/noa).
