# Supplementary material for: Beyond cell cycle control: CDKN2A loss is associated with altered NAD+ metabolic states and increased sensitivity to NAMPT inhibition in glioblastoma
Source: Neurooncol Adv. 2026 Apr 13;8(1):vdag088. doi: 10.1093/noajnl/vdag088 (PMC13092126; doi:10.1093/noajnl/vdag088)
Supplement: vdag088_Supplementary_Data [file vdag088_supplementary_data.docx]

**Materials and Methods**

*Cell lines and culture conditions*

U251 (adult glioblastoma) and SF188 (pediatric high-grade glioma) were cultured in DMEM/F-12, GlutaMAX™ supplement (Gibco™ # 10565018) added with 10% FBS. JHH-NF1-GBM1 (NF1-associated glioblastoma line) and all the GS lines (Patient Derived GBM Cell Lines) were maintained in DMEM/F12, HEPES (ThermoFisher # 11330057) containing 2mM GlutaMax (Gibco™ #35050061), 1X B27 supplement (Gibco™ #12587010), 20 ng/mL EGF (PeproTech, Cranbury, NJ, USA), 20 ng/mL FGF-b (PeproTech), 5 µg/mL Heparin (Millipore SIGMA, Burlington, MA, USA) and 1X Pen-Strep (ThermoFisher # 15140122). TM31 (NF1-associated astrocytoma) was grown in MEM (Gibco #11095080) with 10% FBS, and normal human astrocytes (NHA) were cultured in DMEM (Gibco #11995065) with 2 mM GlutaMAX and 1X Pen-Strep supplemented with 10% FBS. All cultures were maintained at 37°C, 5% CO_2_. Different culture media were used for distinct cell line categories (e.g., serum-containing DMEM-based media for U251, SF188, TM31, and NHA, versus growth factor supplemented neural stem cell media for GS lines and JHH-NF1-GBM1). These media are standard and required to maintain the phenotypic and molecular characteristics of each model. Otherwise, all comparisons within genotype-defined groups were performed under internally consistent culture conditions. Cell identity was confirmed by STR profiling, and mycoplasma testing was performed routinely.

*Cell viability and dose-response curve*

PrestoBlue assay was used to assess cell viability which measures cellular metabolic activity via resazurin reduction. Optimal seeding densities were 1,000 cells/well for U251, SF188, GS lines, and NHA, and 10,000 cells/well for JHH-NF1-GBM1 in 96-well plates. The higher cell density chosen for JHH-NF1-GBM1 (10,000 cells/well) is due to its significantly slower growth kinetics, as previously shown in our earlier report (PMID: 35740680). Cells were plated in 100 µL of culture medium and kept in incubator for 12-16 hours prior to drug treatment. Serial dilutions of candidate compounds GNE-617 and FK866 were added (final volume 200 µL/well) and incubated for five days. The final concentration of DMSO in all treatment conditions was maintained below 0.1% (v/v) and used as vehicle control. Following incubation, 20 µL of PrestoBlue reagent was added, and fluorescence (excitation/emission: 560/590 nm) was measured after 2 hours using Molecular Devices SpectraMax M5 microplate reader. Viability was normalized to DMSO controls, and dose-response curves were generated using nonlinear regression and the AUC were calculated using Graphpad Prism. Cell viability across all cell lines under the treatment of GNE-617 and FK-866 were stratified based on CDKN2A genotype: non-deletion, shallow deletion, and deep deletion. Data was analyzed using mixed-effects models and Benjamini-Hochberg correction for pairwise comparisons. In detail, viability measurements were analyzed in R (v4.4.2) using linear mixed-effects modeling (packages used: lme4, lmerTest, and emmeans). Genotype labels were stratified into four categories (noncancer, ND, SD, DD), and viability was treated as numeric. To reduce the plate/replicate baseline differences and focus inference on drug effects, viability was normalized within each cell line x replicate by the mean viability at 0nM (when available), which yields a relative viability measure. For dose-response modeling, only positive doses (d >0) were analyzed as 0nM baseline dosage was used for normalization and modeled as log_10_(d). The response was clamped to [10^-4^, 1 - 10^-4^] and then logit-transformed. Fixed effects included genotype (noncancer as reference) and a quadratic function of log-dose(log_10_(d) and log_10_(d)^2^). A random intercept per cell line x replicate experimental unit was included to account for correlation among repeated dose measurements within the same experimental unit. A reduced model assumed a common dose-response shape across genotypes, while the full model included genotype x log-dose and genotype x log-dose^2^ interaction terms which allows for genotype-specific curve shapes. The likelihood ratio test comparing the two models provided an omnibus test of whether dose-response relationships differed by genotype. For dose-specific interpretation, model-based estimated marginal means (EMMs) were computed for each genotype at prespecified doses (0.5, 1, 2, 4, 5, 6, 8 and 10 nM) using emmeans, and estimates were back transformed from the logit scale to the relative viability scale using the inverse-logit function. Multiple-comparison control was applied to these post-hoc, dose-wise contrasts using Benjamini-Hochberg FDR, both within each dose (across genotype comparisons at that dose) and across all contrasts pooled over doses.

Apoptosis Assay

U251 cells were seeded at 5,000 cells per well in 96-well plates and allowed to adhere overnight. The following day, cells were treated with FK866 at the indicated concentrations for 24 hours. After treatment, apoptosis was assessed using the Apoptosis/Necrosis Detection Kit (ab176750, Abcam) according to the manufacturer’s instructions.

*In silico analysis*

All analyses were conducted in R (v. 4.3.2). TCGA-GBM gene-level CNV and RNA-seq data were retrieved using TCGAbiolinks R package (PMID: 26704973). CNV data generated through the ABSOLUTE LiftOver workflow were used to define CDKN2A alteration status. Samples with a maximum copy number of 0 were classified as CDKN2A deep-deletion group (DD), whereas those with copy number ≥ 2 were classified as non-deletion (ND). We then matched RNA-seq count data with the grouping method and ran DESeq2 (v. 1.4.6.0) (PMID: 25516281). Gene-set enrichment analyses (PMID: 16199517; 26771021) of DD vs. ND gene sets were performed to identify pathways affected by CDKN2A loss.

*Code availability*

Scripts used to produce figures presented in this manuscript can be downloaded from GitHub at [https://github.com/EdgeYu97/CDKN2A-GBM-Analysis](https://urldefense.com/v3/__https:/github.com/EdgeYu97/CDKN2A-GBM-Analysis__;!!F9wkZZsI-LA!AAIXzRKEE72NLkRhAQohZyi8C0itkAbqVVc2L7cAfeTyQ-W2rAYytgnbAYwkjFTLd75n_xXih6xy-JYp94FYITm50Q$)

**Results**

***Supplementary Figures***

**
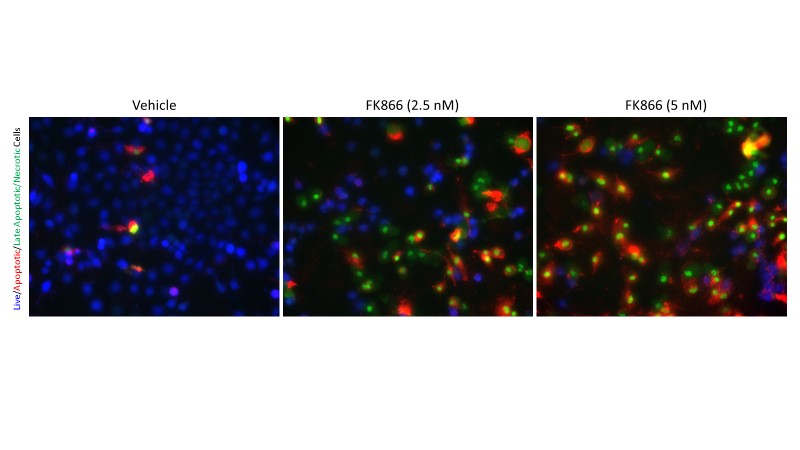
**

**Figure S1** Microscopic images of U251 cells post 24 h treatment with NAMPT inhibitor FK866 at different concentrations. (Live cells: blue (CytoCalcein Violet 450); Apoptotic cells: red (Apopxin Deep Red); Late apoptotic/Necrotic cells: green (Nuclear Green DCS1))


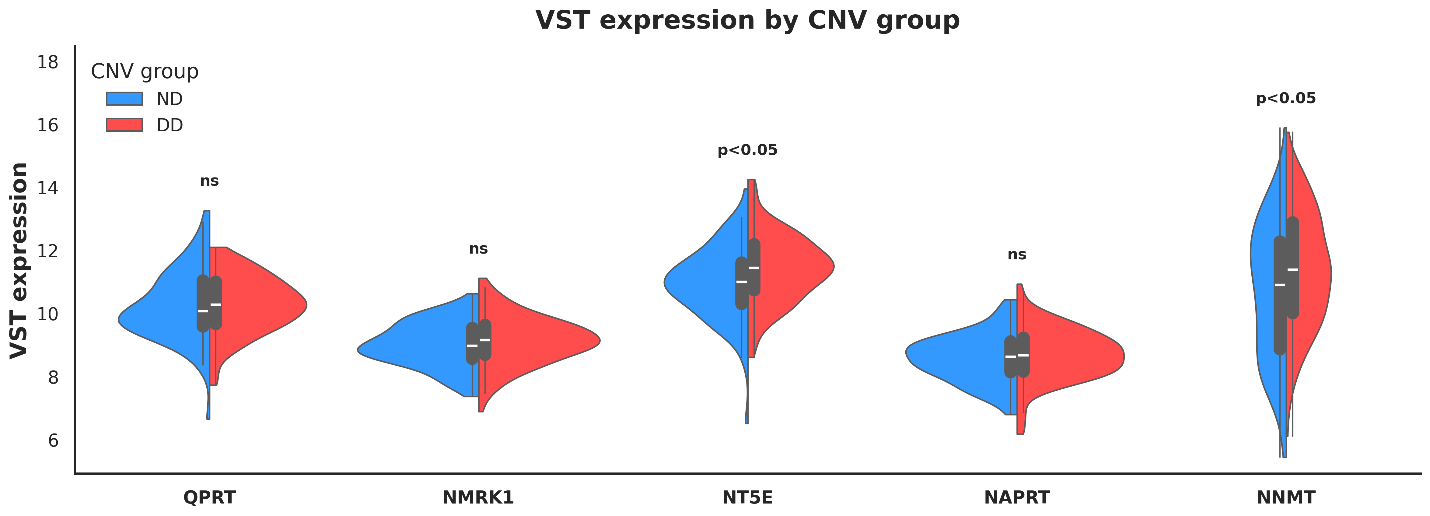


**Figure S2** Variance stabilizing transformation (VST) expression of genes by *CDKN2A* status in TCGA-GBM: non-deletion (ND, blue; copy number ≥2, n=78) vs deep-deletion (DD, red; copy number = 0, homozygous deletion, n=147). Gene-level comparisons were adjusted for multiple testing using FDR correction.

***Supplementary Tables***

**Table S1** Per Cell Line Area Under the Curve (AUC) Summary Metrics for NAMPT Inhibitors (GNE-617 and FK866) Sensitivity Across GBM Models

| **Cell Line** | **CDKN2A status** | ***AUC (GNE-617)** | ***AUC (FK866)** |
| --- | --- | --- | --- |
| GS243 | No deletion | 5.871 ± 0.15 | 5.331 ± 0.13 |
| GS208 | No deletion | 6.470 ± 0.11 | 7.337 ± 0.15 |
| GS142 | No deletion | 5.099 ± 0.15 | 6.397 ± 0.11 |
| GS116 | No deletion | 8.102 ± 0.13 | 7.787 ± 0.09 |
| GS104 | No deletion | 2.685 ± 0.05 | 3.908 ± 0.05 |
| GS183 | Shallow Deletion | 5.467 ± 0.11 | 6.530 ± 0.11 |
| GS187 | Deep Deletion | 6.848 ± 0.13 | 5.817 ± 0.12 |
| GS025 | Deep deletion | 1.312 ± 0.03 | 1.023 ± 0.02 |
| GS075 | Deep Deletion | 4.584 ± 0.10 | 4.578 ± 0.08 |
| JHH-NF1-GBM1 | Deep Deletion | 3.799 ± 0.05 | 3.906 ± 0.06 |
| TM31 | Deep Deletion | 2.622 ± 0.10 | 5.226 ± 0.07 |
| SF188 | Deep Deletion | 2.090 ± 0.04 | 3.142 ± 0.03 |
| U251 | Deep Deletion | 1.876 ± 0.03 | 6.087 ± 0.09 |

*Area ± SE

**Table S2** Results of mixed-effects models and Benjamini-Hochberg correction for pairwise comparisons for GNE-617

| **GNE-617 dose_nM** | **contrast** | **p.value** | **padj_BH_within_dose** | **sig_within** | **padj_BH_all** | **sig_pooled** |
| --- | --- | --- | --- | --- | --- | --- |
| 0.5 | ND - DD | 0.080852593 | 0.121278889 | ns | 0.107803457 | ns |
| 0.5 | ND - SD | 0.071795174 | 0.121278889 | ns | 0.101357892 | ns |
| 0.5 | SD - DD | 0.761439825 | 0.761439825 | ns | 0.891441746 | ns |
| 0.5 | noncancer - DD | 0.00883703 | 0.02651109 | * | 0.014139248 | * |
| 0.5 | noncancer - ND | 0.264060776 | 0.316872932 | ns | 0.324997878 | ns |
| 0.5 | noncancer - SD | 0.006124639 | 0.02651109 | * | 0.010137333 | * |
| 1 | ND - DD | 0.000748007 | 0.001173953 | ** | 0.001329791 | ** |
| 1 | ND - SD | 0.103653482 | 0.124384178 | ns | 0.134469382 | ns |
| 1 | SD - DD | 0.999102046 | 0.999102046 | ns | 0.999995459 | ns |
| 1 | noncancer - DD | 2.2586E-07 | 1.35516E-06 | *** | 6.77581E-07 | *** |
| 1 | noncancer - ND | 0.000782636 | 0.001173953 | ** | 0.001341661 | ** |
| 1 | noncancer - SD | 2.51148E-05 | 7.53445E-05 | *** | 5.66466E-05 | *** |
| 2 | ND - DD | 0.000140213 | 0.000210319 | *** | 0.000269208 | *** |
| 2 | ND - SD | 0.442883075 | 0.531459691 | ns | 0.531459691 | ns |
| 2 | SD - DD | 0.803685638 | 0.803685638 | ns | 0.918497872 | ns |
| 2 | noncancer - DD | 3.81971E-10 | 2.29183E-09 | *** | 2.01458E-09 | *** |
| 2 | noncancer - ND | 2.66459E-06 | 5.32918E-06 | *** | 6.73159E-06 | *** |
| 2 | noncancer - SD | 1.90842E-06 | 5.32918E-06 | *** | 5.08913E-06 | *** |
| 4 | ND - DD | 3.85627E-05 | 5.78441E-05 | *** | 8.04787E-05 | *** |
| 4 | ND - SD | 0.928255786 | 0.928255786 | ns | 0.999995459 | ns |
| 4 | SD - DD | 0.18398729 | 0.220784748 | ns | 0.232404997 | ns |
| 4 | noncancer - DD | 3.63561E-10 | 2.18136E-09 | *** | 2.01458E-09 | *** |
| 4 | noncancer - ND | 3.23184E-09 | 9.69551E-09 | *** | 1.41026E-08 | *** |
| 4 | noncancer - SD | 1.24705E-07 | 2.49409E-07 | *** | 3.99055E-07 | *** |
| 5 | ND - DD | 2.5963E-05 | 3.89446E-05 | *** | 5.66466E-05 | *** |
| 5 | ND - SD | 0.992660665 | 0.992660665 | ns | 0.999995459 | ns |
| 5 | SD - DD | 0.078510988 | 0.094213186 | . | 0.107672212 | ns |
| 5 | noncancer - DD | 1.07403E-10 | 6.44416E-10 | *** | 1.28883E-09 | *** |
| 5 | noncancer - ND | 4.19705E-10 | 1.25911E-09 | *** | 2.01458E-09 | *** |
| 5 | noncancer - SD | 6.07699E-08 | 1.2154E-07 | *** | 2.24381E-07 | *** |
| 6 | ND - DD | 2.29325E-05 | 3.43987E-05 | *** | 5.5038E-05 | *** |
| 6 | ND - SD | 0.999995459 | 0.999995459 | ns | 0.999995459 | ns |
| 6 | SD - DD | 0.03686833 | 0.044241996 | * | 0.053626662 | . |
| 6 | noncancer - DD | 9.48978E-11 | 4.49422E-10 | *** | 1.28883E-09 | *** |
| 6 | noncancer - ND | 1.49807E-10 | 4.49422E-10 | *** | 1.43815E-09 | *** |
| 6 | noncancer - SD | 4.45895E-08 | 8.9179E-08 | *** | 1.78358E-07 | *** |
| 8 | ND - DD | 4.02608E-05 | 6.03913E-05 | *** | 8.05217E-05 | *** |
| 8 | ND - SD | 0.958753482 | 0.958753482 | ns | 0.999995459 | ns |
| 8 | SD - DD | 0.013816966 | 0.016580359 | * | 0.020725449 | * |
| 8 | noncancer - DD | 2.37976E-10 | 9.06767E-10 | *** | 1.9038E-09 | *** |
| 8 | noncancer - ND | 3.02256E-10 | 9.06767E-10 | *** | 2.01458E-09 | *** |
| 8 | noncancer - SD | 7.00596E-08 | 1.40119E-07 | *** | 2.40204E-07 | *** |
| 10 | ND - DD | 0.000171799 | 0.000257698 | *** | 0.000317167 | *** |
| 10 | ND - SD | 0.846668377 | 0.846668377 | ns | 0.945118188 | ns |
| 10 | SD - DD | 0.01055512 | 0.012666144 | * | 0.016343412 | * |
| 10 | noncancer - DD | 4.06342E-14 | 2.43805E-13 | *** | 1.95044E-12 | *** |
| 10 | noncancer - ND | 1.72269E-11 | 5.16807E-11 | *** | 4.13445E-10 | *** |
| 10 | noncancer - SD | 3.75218E-07 | 7.50436E-07 | *** | 1.05944E-06 | *** |
| null | null | null | null | null | null | null |

**Table S3** Results of mixed-effects models and Benjamini-Hochberg correction for pairwise comparisons for FK866

| **FK866**  **dose_nM** | **contrast** | **p.value** | **padj_BH_within_dose** | **sig_within** | **padj_BH_all** | **sig_pooled** |
| --- | --- | --- | --- | --- | --- | --- |
| 0.5 | ND - DD | 0.47807487 | 0.717112306 | ns | 0.637433161 | ns |
| 0.5 | ND - SD | 0.714967739 | 0.857961287 | ns | 0.879960294 | ns |
| 0.5 | SD - DD | 0.99999962 | 0.99999962 | ns | 0.99999962 | ns |
| 0.5 | noncancer - DD | 0.021136629 | 0.112832421 | ns | 0.039021468 | * |
| 0.5 | noncancer - ND | 0.134948611 | 0.269897222 | ns | 0.202422917 | ns |
| 0.5 | noncancer - SD | 0.037610807 | 0.112832421 | ns | 0.064475669 | . |
| 1 | ND - DD | 0.141675711 | 0.212513566 | ns | 0.206073761 | ns |
| 1 | ND - SD | 0.658476692 | 0.79017203 | ns | 0.831760032 | ns |
| 1 | SD - DD | 0.999666024 | 0.999666024 | ns | 0.99999962 | ns |
| 1 | noncancer - DD | 6.14527E-08 | 3.68716E-07 | *** | 1.63874E-07 | *** |
| 1 | noncancer - ND | 1.70225E-05 | 3.4045E-05 | *** | 3.89086E-05 | *** |
| 1 | noncancer - SD | 1.30955E-05 | 3.4045E-05 | *** | 3.14293E-05 | *** |
| 2 | ND - DD | 0.079375227 | 0.119062841 | ns | 0.122903578 | ns |
| 2 | ND - SD | 0.88306849 | 0.952696868 | ns | 0.99999962 | ns |
| 2 | SD - DD | 0.952696868 | 0.952696868 | ns | 0.99999962 | ns |
| 2 | noncancer - DD | 7.88811E-11 | 4.73287E-10 | *** | 4.73287E-10 | *** |
| 2 | noncancer - ND | 3.77566E-08 | 1.1327E-07 | *** | 1.1327E-07 | *** |
| 2 | noncancer - SD | 8.79561E-07 | 1.75912E-06 | *** | 2.22205E-06 | *** |
| 4 | ND - DD | 0.023507859 | 0.035261788 | * | 0.041791749 | * |
| 4 | ND - SD | 0.995801206 | 0.995801206 | ns | 0.99999962 | ns |
| 4 | SD - DD | 0.531730615 | 0.638076738 | ns | 0.689812689 | ns |
| 4 | noncancer - DD | 1.35336E-13 | 8.12017E-13 | *** | 1.22569E-12 | *** |
| 4 | noncancer - ND | 2.01599E-10 | 6.04796E-10 | *** | 1.07519E-09 | *** |
| 4 | noncancer - SD | 4.88662E-08 | 9.77324E-08 | *** | 1.37975E-07 | *** |
| 5 | ND - DD | 0.009004073 | 0.013506109 | * | 0.017287819 | * |
| 5 | ND - SD | 0.999983049 | 0.999983049 | ns | 0.99999962 | ns |
| 5 | SD - DD | 0.314221204 | 0.377065445 | ns | 0.430931937 | ns |
| 5 | noncancer - DD | 5.24025E-14 | 3.14415E-13 | *** | 8.3844E-13 | *** |
| 5 | noncancer - ND | 8.95373E-12 | 2.68612E-11 | *** | 6.1397E-11 | *** |
| 5 | noncancer - SD | 8.64348E-09 | 1.7287E-08 | *** | 2.76591E-08 | *** |
| 6 | ND - DD | 0.003508863 | 0.005263294 | ** | 0.007017725 | ** |
| 6 | ND - SD | 0.999116476 | 0.999116476 | ns | 0.99999962 | ns |
| 6 | SD - DD | 0.176826541 | 0.212191849 | ns | 0.249637469 | ns |
| 6 | noncancer - DD | 4.46041E-10 | 1.39764E-09 | *** | 2.03294E-09 | *** |
| 6 | noncancer - ND | 4.65881E-10 | 1.39764E-09 | *** | 2.03294E-09 | *** |
| 6 | noncancer - SD | 2.27938E-09 | 4.55875E-09 | *** | 7.81501E-09 | *** |
| 8 | ND - DD | 0.001009321 | 0.001513981 | ** | 0.002106409 | ** |
| 8 | ND - SD | 0.977964396 | 0.977964396 | ns | 0.99999962 | ns |
| 8 | SD - DD | 0.069540554 | 0.083448664 | . | 0.111264886 | ns |
| 8 | noncancer - DD | 8.32667E-15 | 4.996E-14 | *** | 3.9968E-13 | *** |
| 8 | noncancer - ND | 1.53211E-13 | 4.59632E-13 | *** | 1.22569E-12 | *** |
| 8 | noncancer - SD | 9.114E-10 | 1.8228E-09 | *** | 3.6456E-09 | *** |
| 10 | ND - DD | 0.000937336 | 0.001406003 | ** | 0.002045096 | ** |
| 10 | ND - SD | 0.943541318 | 0.943541318 | ns | 0.99999962 | ns |
| 10 | SD - DD | 0.045678083 | 0.0548137 | . | 0.075605103 | . |
| 10 | noncancer - DD | 8.90399E-14 | 2.6712E-13 | *** | 1.06848E-12 | *** |
| 10 | noncancer - ND | 1.75415E-14 | 1.05249E-13 | *** | 4.20997E-13 | *** |
| 10 | noncancer - SD | 1.42414E-09 | 2.84828E-09 | *** | 5.25836E-09 | *** |
| null | null | null | null | null | null | null |
